# Immunodeficiency and Autoimmunity: The goals of the Journal of Translational Autoimmunity

**DOI:** 10.1016/j.jtauto.2019.01.001

**Published:** 2019-01-17

**Authors:** Christopher Chang, M. Eric Gershwin

**Affiliations:** a Division of Rheumatology, Allergy and Clinical Immunology, University of California, Davis, Davis, CA 95616, USA; b Division of Pediatric Immunology and Allergy, Joe DiMaggio Children's Hospital, Memorial Health System, Hollywood, FL 33021, USA; c Department of Pediatrics, Florida Atlantic University, Boca Raton, FL 33431, USA

The primary objective of the Journal of Translational Autoimmunity, a new peer-reviewed medical journal, is the sharing of scientific information related to autoimmunity. The focus of the Journal is to address the mechanisms and pathogenesis that leads to a loss of tolerance that is the common endpoint in patients with autoimmunity. The essence of translational medicine is to link the laboratory with the clinic, the premise being a better understanding of the pathophysiology behind the disease allows for better diagnosis and treatment, and therefore, better patient care. This is a mechanistic approach to medicine. The Journal will provide contributors with different platforms on which to share their work, ranging from original research papers, to reviews, case studies or reports, and short communications. All papers will be peer reviewed by an expert group of scientist and physicians carefully selected by the Editors and the Editorial Board, to ensure that each publication is of the highest quality and scientific merit.

The scope of this new Journal includes translational research on a variety of autoimmune diseases, including but not limited to systemic lupus erythematosus, discoid and cutaneous lupus, psoriasis, Sjogren's syndrome, autoimmune hepatitis, type 1 diabetes, juvenile idiopathic arthritis, rheumatoid arthritis, autoimmune cytopenias, idiopathic thrombocytopenia, primary biliary cholangitis, anti-phospholipid syndrome and dermatomyositis. It has been said before that autoimmunity is a result of “bad genes and bad luck”. But a greater understanding of the mechanisms of disease and the effect of these pathophysiologic changes on the clinical presentation of autoimmune diseases may allow us in the future to mitigate the effects of “bad genes” and overcome the unpredictability of “bad luck”. It is translational medicine that will eventually allow us to fulfill the dream of personalized medicine, whereupon we will be able to match the best treatment with the right patient.

In order to adequately address this group of disorders, one must also recognize their co-existence with another group of diseases, the primary immunodeficiencies. The relationship between immunodeficiency diseases and autoimmunity is a prime example of how understanding of the complex pathways that allow for the co-existence of two seemingly polar opposites of hypo- and hyper-reactive immune systems can assist us in formulating optimal treatment regimens for these patients.

Since the discovery of Bruton's Agammaglobulinemia some 66 years ago, over 350 primary immunodeficiency diseases have been identified. Some of these are monogenic while others may be multifactorial. However, even monogenic immunodeficiencies do not live in a vacuum. In fact, most of the immunodeficiencies are accompanied by other associated diseases, including autoimmune diseases, allergic diseases and cancer.

Immunodeficiency diseases result in an inability to fight infections and other factors that can cause damage to the body. This is intuitive, as the word “deficiency” usually signifies missing something, as in a crucial factor that leads to some form of decreased immunity or protection. Since the immune system is involved in surveillance for diseases, it is also intuitive that certain immune defects may result in some tumor cells or danger signals being missed, and thereby lead to malignancy. Conversely, autoimmune diseases and allergies are generally thought to result from a hyperimmune state. How is it that an immunodeficiency can be associated with conditions of immune hyperreactivity? [1].

It is clear from the knowledge gained from the previous decades that immunodeficiency is not as simple as a missing cog in immune physiology. The complexity of the immune system has taught us that everything is interconnected, and that one missing factor can in fact lead to changes in various pathways that can lead to a simultaneous depression of certain parts of the immune system and increase in activity in other areas. The immune system doesn't just protect, it also regulates what is viewed as self and what should be allowed to coexist within our bodies without harm. It is this balance that is disrupted in the many diseases classified as immunodeficiencies. In fact, most “immunodeficiencies” can really be more accurately described as “immunodysfunctions”.

An example of how this can occur can be found in the soluble IL2 receptor alpha molecule, or CD25. CD25 is critical to the activity of Treg cells, but it is also present on a variety of T cells. It is now known that one of the mechanisms for the development of autoimmunity may be the balance between Th17 cells and Treg cells to maintain immune homeostasis. When this balance is disrupted, it allows for the breakdown of tolerance. A genetic mutation that leads to a defect in CD25 will affect not just T helper and cytotoxic T cells that are involved in protection against infection, but Treg cells as well. These different cell types are potentially both affected, albeit to different degrees, and the presence or absence of alternative pathways to maintain the respective populations and/or activities of various immune cell types can simultaneously lead to a variety of autoimmune and immunodeficient phenotypes. This complex interrelationship of factors repeats itself in many other genetic immunodeficiencies.

Nearly all the immunodeficiencies, no matter where the defect lies, are associated with autoimmunity. Humoral immunodeficiencies such as selective IgA deficiency (sIgAD) are associated with a variety of autoimmune diseases [2]. HyperIgM syndrome is also associated with the presence of autoantibodies found in a variety of autoimmune diseases, including primary biliary cholangitis [3]. Mixed B and T cell defects that lead to combined variable immunodeficiency (CVID) are associated with autoimmune cytopenias. CVID may be associated with a greater risk for malignancy, especially lymphoma, as well as interstitial lung diseases, such as lymphocytic interstitial pneumonia (LIP) or granulomatous-lymphocytic interstitial lung disease (GLILD) [4], which can result from related defects in immune function, that may in turn be related to human herpesvirus-8 (HHV-8) infection [5]. Complement deficiencies such as C1q, C2 and C4 deficiencies are all associated with a variable higher risk for systemic lupus erythematosus.

In autoimmune polyendocrinopathy, candidiasis and ectodermal dystrophy (APECED) or autoimmune polyendocrinopathy syndrome type 1 (APS-1), mutations in the *AIRE* gene leads to autoreactive T cells in the thymus being able to evade the normal negative selection of self-reactive T cells, which leads to a defect in central tolerance [6]. This leads to a multitude of autoimmune problems that primarily affect the endocrine system, including hypoparathyroidism and adrenal failure. From an immunodeficiency standpoint, these patients present with mucocutaneous candidiasis early in life, and exhibit an overall defective immune response to *Candida* antigens. However, it is still unclear whether the primary defect is in T cell proliferation response against *Candida albicans*, a defect of monocyte intracellular signaling, or an alteration in Th17 immune response.

In the relatively common T cell immunodeficiency syndrome named after Angelo DiGeorge, the genetic mutation typically involves a deletion in the 22q11.2 locus. Of the variable number of genes present within this deletion is *TBX1*, which encodes for a protein named T-box 1, which plays a role in embryonic development of the structures around the neck, including the thymus, muscles and bones, the heart and great vessels, and the parathyroid gland. The resulting immunological defect is variable, but in severe cases, may lead to reduced T cell differentiation and proliferation, resulting in recurrent infections and a concomitant reduction in Treg cells, which may also lead to autoimmune diseases. The incidence of autoimmune disease in DiGeorge Syndrome is about 10%, and ranges from autoimmune cytopenias to Hashimoto's thyroiditis [7]. It is important to note that the association between DiGeorge Syndrome and autoimmune diseases may extend beyond the 22q11.2 deletion as the sole pathophysiology.

There are numerous other examples of associations between primary immunodeficiencies and autoimmunity. The mechanisms that may be in play include molecular mimicry, innocent bystander, cryptic antigens and epitope spreading. Each of these mechanisms may contribute to the link between infections, immunodeficiency and autoimmunity.

Many immunodeficiencies are simultaneously and randomly associated with multiple autoimmune diseases (Fig. 1). Perhaps the next question should be whether or not this phenomenon exists in secondary immunodeficiencies such as human immunodeficiency virus (HIV), or whether acquired immunodeficiency accompanies the development of autoimmune diseases, and if the mechanisms are the same. In the meantime, we think that the term “Primary immunodeficiency diseases” should be changed to “Primary immune dysfunction syndrome” in cases where immunodeficiency and autoimmunity coexist, and that there should be a separate subspecialty dedicated to the study of these diseases that cross multiple disciplines.

**Fig. 1:**
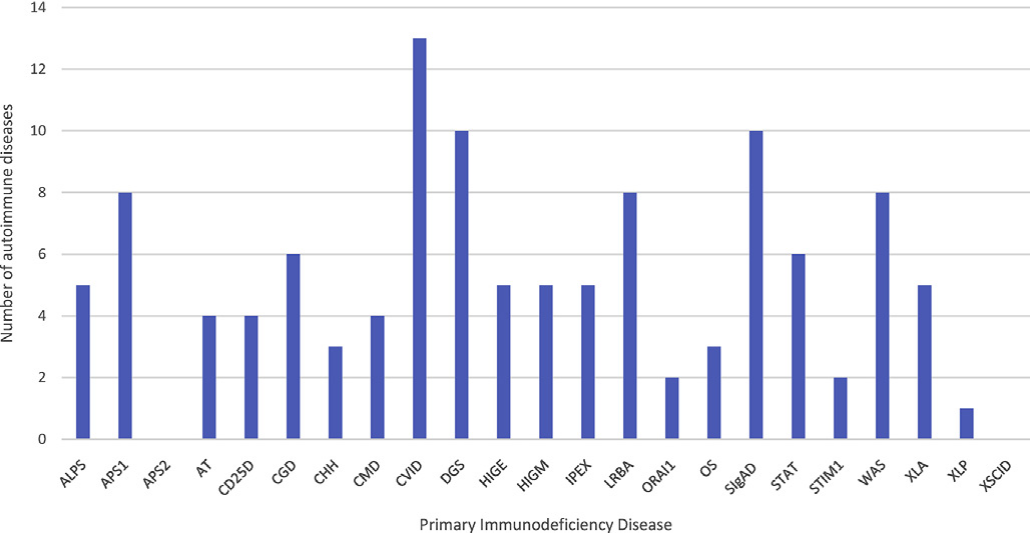
**Association of autoimmune diseases and primary immunodeficiency diseases.** The number of different autoimmune diseases reported for each primary immunodeficiency disease. The x-axis represents the primary immunodeficiency. More and more autoimmune diseases are being reported to occur in primary immunodeficiency diseases. This data is updated as of 12/18/2018.

## Abbreviations

**Primary Immunodeficiency Diseases (PID)**
ALPS: Autoimmune lymphoproliferative syndrome
APS1: Autoimmune polyendocrine syndrome, type 1 (APECED)
APS2: Autoimmune polyendocrine syndrome, type 2
AT: Ataxia telangiectasia
CD25D: CD25 deficiency
CGD: Chronic granulomatous disease
CHH: Cartilage-hair hypoplasia
CMD: Complement deficiency
CVID: Common variable immunodeficiency
DGS: DiGeorge syndrome
HIGE: Hyper IgE syndrome
HIGM: Hyper IGM syndrome
IPEX: Immune dysregulation, polyendocrinopathy, enteropathy, X-linked
LRBA: Lipopolysaccharide responsive beige-like anchor
OS: Omenn Syndrome
sIgAD: selective IgA deficiency
STAT: signal transducer and activator of transcription
STIM1: Stromal interaction molecule 1
WAS: Wiskott-Aldrich syndrome
XLA: X-linked agammaglobulinemia
XLP: X-linked lymphoproliferative disease
XSCID: X-linked severe combined immunodeficiency
